# Persistently high SARS-CoV-2 positivity rate and incidence for Hispanic/Latinos during state reopening in an urban setting: a retrospective cohort study

**DOI:** 10.1017/S0950268821000133

**Published:** 2021-01-18

**Authors:** Chien-Hsiang Weng, Andrew Saal, Daniel C. McGuire, Philip A. Chan

**Affiliations:** 1 Brown University Warren Alpert Medical School, Providence, Rhode Island 02903, USA; 2 Providence Community Health Centers, Providence, Rhode Island 02905, USA

**Keywords:** COVID-19, Hispanic, positivity rate, reopening, SARS-CoV-2, urban

## Abstract

Hispanic/Latino populations are disproportionately impacted by coronavirus disease 2019 (COVID-19) in the United States. The impact of state reopening on COVID-19 in this population after stay-at-home orders is unknown. We evaluated the incidence, prevalence and trends during reopening of severe acute respiratory syndrome-coronavirus-2 (SARS-CoV-2) at a major federally qualified health centre in Providence, Rhode Island. A total of 14 505 patients were tested for SARS-CoV-2 from 19 March to 18 August 2020, of which, data on 13 318 (91.8%) patients were available; 70.0% were Hispanic/Latino, and 2905 were positive for SARS-CoV-2 infection. The urban Hispanic/Latino population was almost five times more likely to test positive for SARS-CoV-2 (risk ratio 4.97, 95% CI 2.59–9.53, *P* < 0.001) compared to non-Hispanic White. The positivity rates among the urban Hispanic/Latino population remained >10% during all phases of reopening. The trends of the incidence rates showed similar associations to those we observed for positivity rates. Public health interventions to address SARS-CoV-2 in Hispanic/Latino communities are urgently needed, even in latter phases of state reopening.

Coronavirus disease 2019 (COVID-19), caused by severe acute respiratory syndrome coronavirus-2 (SARS-CoV-2), has resulted in a worldwide pandemic including over 19.8 million cases in the United States as of 31 December 2020 [1]. In the US, Hispanic/Latino populations are disproportionately impacted by SARS-CoV-2 [2, 3]. To address the pandemic, many states have implanted stay-at-home orders to limit the spread of the virus in the community [4]. As the pandemic has progressed, many states have gradually opened during several ‘phases’ which have included a loosening of restrictions on work and movement. Data on the impact of phased reopening on cases of SARS-CoV-2 in populations at higher-risk, including Hispanic/Latinos, are limited.

In the State of Rhode Island (RI), Hispanic/Latino individuals are up to three times more likely to test positive for SARS-CoV-2 [2]. The positivity rate of SARS-CoV-2 testing is an important measure in evaluating the spread and control of the disease. It is a relative approximation of how prevalent the virus is and is also dependent on the overall testing volume. States are using this to make policy decisions about quarantining visitors from other areas as well as to determine sufficient testing capacity. To address COVID-19 in Rhode Island, an initial stay-at-home order was implemented on 28 March 2020. Peak COVID-19 cases were observed in late April. Subsequently, the state underwent a series of phased re-opening stages according to the disease trend [5]. Phase 1 was to lift the stay-at-home order and allow social gatherings up to 10 people (9 May 2020), while phase 2 allowed up to 15 people (1 June 2020) and phase 3 up to 50 people. During each phase, different settings were allowed back to work including restaurants, public beaches and other settings.

To understand how reopening affected SARS-CoV-2 cases, with a focus on Hispanic/Latinos, we reviewed the data from a major federally qualified health centre (FQHC) in Providence, Rhode Island, which consisted of 10 neighborhood clinics and approximately 60 000 patients or one third of the total population in Providence. FQHCs are community health centres which receive federal funding to provide primary care services in underserved areas. Providence is the largest urban centre in the state and consistently the town/city with the greatest number of reported SARS-CoV-2 cases. We examined the number of COVID-19 cases and SARS-CoV-2 per cent positivity over time across different race/ethnicity populations and determined the overall positivity rate (number positive/number tested) and incidence rate (number positive/population covered) per week. The patient population at the FQHC is predominantly Hispanic/Latino and 90% of households are under 200% federal poverty level (FPL), which is a measure of income issued every year by the Department of Health and Human Services. Different FPLs are used to determine the eligibility for certain programmes and benefits. Every patient of this FQHC who was tested for SARS-CoV-2 by reverse-transcription polymerase chain reaction (RT-PCR) was included in this study from 19 March 2020. We characterised patients by demographics, insurance status and SARS-CoV-2 test results. If a patient had multiple positive tests, only the first test was counted for the purpose of this analysis. We reported numbers (percentages) for binary/categorical variables and medians (interquartile ranges, IQR) for continuous variables. Chi-square tests and Wilcoxon rank-sum tests were applied to determine statistically significant differences among groups. A two-sided significance threshold was set at *P* < 0.05. The Providence Community Health Centers Review Committee approved the project. All analyses were run using STATA 13.1 (StataCorp, College Station, TX).

A total of 14 505 patients were tested for SARS-CoV-2 from 19 March to 18 August 2020, of which, data on 13 318 (91.8%) patients were available; 65.9% were female, 70.0% were self-reported as Hispanic/Latino and 85.3% were insured. Overall, 11 274 individuals had one test, 1871 had two tests and 173 had three or more (Table 1). Of the Hispanic/Latino patients tested, 25.5% (2832/9328) were positive, compared to 15.7% (172/1095) of non-Hispanic (NH) Blacks, 6.6% (58/873) of NH Whites and 14.5% (293/2022) of NH others (*P* < 0.001). A total of 82.0% of the total positive tests were from Hispanic/Latino patients, 6.1% from NH Black, 2.1% from NH White and 9.8% from NH others. The Hispanic/Latino population experienced a significant decrease in the positivity rates between phases 1 and 3 (22.8−12.1%, respectively). This was in comparison to the NH White population which experienced a 2.5% reduction and NH Blacks which experienced a 13.1% reduction. When comparing to statewide trends, Rhode Island experienced a 3.5% reduction in cases over the phased reopening with a steadily low positivity rate during phase 3, similar to the NH White population at our clinic. Despite this, the positivity rate among the Hispanic/Latino population was still >10% even during phase 3 (Fig. 1). During the entire period, the Hispanic/Latino population was almost five times more likely to test positive for SARS-CoV-2 (risk ratio 4.97, 95% CI 2.59–9.53, *P* < 0.001) compared to NH White, and 2.6 times more likely compared to NH Black (risk ratio 2.61, 95% CI 1.70–4.00, *P* < 0.001). The subgroup analysis by their insurance status, which may reflect the socioeconomic or legal status, we found much higher positivity rates in all race/ethnicity groups who were uninsured except the NH Blacks. Uninsured Hispanic/Latino patients were still 2.6 and 2.4 times more likely to test positive for SARS-CoV-2 compared to the uninsured NH White and uninsured NH Black, respectively (risk ratio 2.59, 95% CI 1.41–4.75, *P* < 0.001; risk ratio 2.38, 95% CI 1.55–3.66, *P* < 0.001). The positivity rate in NH Black population has dropped below 10% during phase 3. However, when compared to the NH White population, they were still two times more likely to test positive (risk ratio 2.11, 95% CI 1.59–2.81, *P* < 0.001). A total of 21.2% Hispanic/Latino patients tested positive at the FQHC during the entire study period, which was significantly higher than the state average (4.7%) as of 22 August 2020 [6]. Comparing to the statewide positivity rate, our Hispanic/Latino population was 4.5 times more likely to test positive for SARS-CoV-2 (risk ratio 4.51, 95% CI 4.34–4.68, *P* < 0.001), while NH Black population was 2.7 times (risk ratio 2.72, 95% CI 2.36–3.13, *P* < 0.001), and NH White was 1.29 times (95% CI 1.00–1.65, *P* = 0.048).

**Fig. 1.**
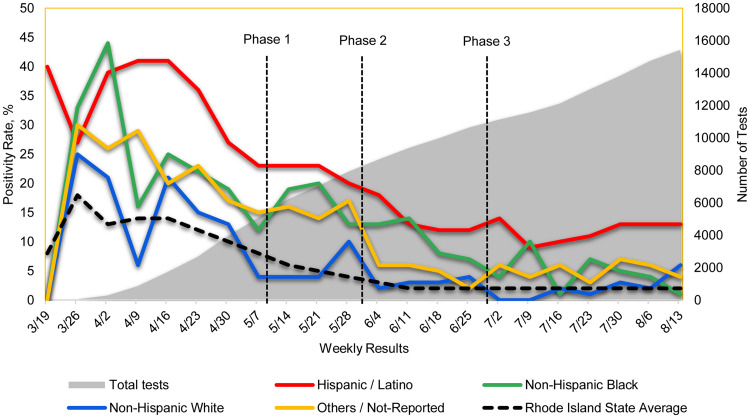
SARS-CoV-2 positivity rates at Providence Community Health Centers *vs*. the Rhode Island State average. The red line represents the per cent positivity of the Hispanic/Latino population over reopening phases 1−3 compared to other populations. The per cent positivity of the Hispanic/Latino population never dropped below 10%. The shaded grey area represents the total number of cumulative tests over time.

**Table 1.** Patient characteristics and results of SARS-CoV-2 testing

	SARS-CoV-2 tested individuals at PCHC (*N* = 13 318)			SARS-CoV-2 tested individuals in Rhode Island (*N* = 224 929)	
	Positive	Negative	*P* value	Positive	Negative
Total – individual (%)	2905 (21.8)	10 413 (78.2)		19 880 (8.8)	205 049 (91.2)
Sex – no. (%)					
Female	1830 (63.0)	6947 (66.7)	<0.001	10 963 (55.1)	110 985 (54.1)
Male	1075 (37.0)	3464 (33.3)		8826 (44.4)	87 300 (42.6)
Pending further information	0	2 (<1)		91 (<1)	6764 (3.3)
Median age − years (IQR)	37.0 (26.0-49.0)	35.0 (25.0-50.0)	0.178	–	–
Race/Ethnicity – no. (%)					
Hispanic/Latino	2382 (82.0)	6946 (66.7)	<0.001	7274 (36.6)	12 672 (6.2)
Non-Hispanic Black	172 (5.9)	923 (8.9)		1896 (9.5)	4987 (2.4)
Non-Hispanic White	58 (2.0)	815 (7.8)		5655 (28.4)	38 177 (18.6)
Others (including race/ethnicity unknown)^a^	293 (10.1)	1729 (16.6)		5055 (25.4)	149 213 (72.8)
Age group (years old) – no. (%)					
0–9	138 (4.7)	620 (6.0)	<0.001	604 (3.0)	6930 (3.4)
10–19	247 (8.5)	738 (7.1)		1235 (6.2)	12 028 (5.9)
20–39	1194 (41.1)	4670 (44.8)		6576 (33.1)	69 482 (33.9)
40–59	1023 (35.2)	3104 (29.8)		5955 (30.0)	60 997 (29.7)
60–79	269 (9.3)	1141 (11.0)		3477 (17.5)	42 996 (21.0)
80+	34 (1.2)	140 (1.3)		2028 (10.2)	12 464 (6.1)
Pending further information				5	157
Insurance status − no. (%)					
Insured	2250 (77.5)	9110 (87.5)	<0.001	–	–
Uninsured	655 (22.5)	1303 (12.5)		–	–

PCHC: Providence Community Health Centers; IQR: interquartile range.

a Others included: Asian, more than one race (non-Hispanic), native Hawaiian, other Pacific islander, race/ethnicity unknown.

We also compared the weekly incidence rates between our Hispanic/Latino, NH Black, NH White populations and the statewide population. According to the United States Census Bureau, there are 179 883 residents in Providence, RI and 1 059 361 in the State of Rhode Island [7]. There are 42 240 Hispanic/Latino patients, 4538 NH Black and 3991 NH White among the communities our FQHC serves. The weekly incidence rates among the Hispanic/Latino population have been more than three times higher than the statewide incidence rates for the majority of the time since the pandemic throughout the reopening phases. NH Black as well had significantly higher incidence until phase 3 reopening while NH White had similar rates throughout the entire period compared to the statewide trend (Fig. 2). The incidence trends were consistent with the trends of the positivity rates in our study. When comparing the incidence of COVID-19 to the statewide average, our Hispanic/Latino population was 3 times higher (incidence rate ratio 3.01, 95% CI 2.88–3.14, *P* < 0.001), while NH Black population was 2 times (incidence rate ratio 2.02, 95% CI 1.73–2.35, *P* < 0.001) and NH White was slightly lower (incidence rate ratio 0.77, 95% CI 0.59–1.00, *P* = 0.045).

**Fig. 2.**
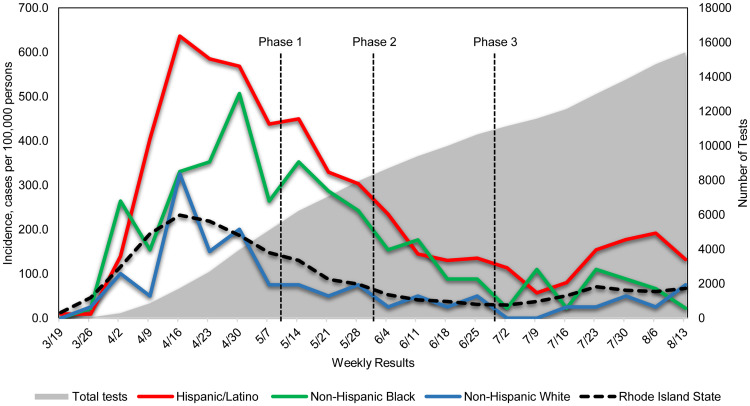
New positive COVID-19 cases and estimated incidence by race/ethnicity group and week. The red line represents the weekly COVID-19 incidence of the Hispanic/Latino population over reopening phases 1−3 compared to other populations. The weekly incidence rates among the Hispanic/Latino population have been significantly higher than the statewide weekly incidence rates since the pandemic throughout the reopening phases. The shaded grey area represents the total number of cumulative tests over time.

This is among the first studies to evaluate changes in rates of SARS-CoV-2 among Hispanic/Latinos over time and during phased reopening, and demonstrates persistently high positivity rates and incidence in this population. Reassuringly, the urban Hispanic/Latino populations did experience a drop in cases over time as did other race/ethnicity groups. However, the positivity rates remained steadily above 10% in the Hispanic/Latino population in phase 3 without further reduction. We suspect that this might be similar in other urban areas. Positivity rates can be used as a marker of both access to testing, and burden of infection in the community. Positivity rates below 5% are generally considered adequate in terms of testing penetration and lower rates of infection [8]. The phased reopening was implemented based on the state-level incidence and positivity trends, which were inconsistent with those observed among the vulnerable population groups included in this study. The persistently higher rate >10% in all phases of reopening suggest that more needs to be done in terms of access to SARS-CoV-2 testing, as well as focused interventions for this community.

The persistently high positive rate could be explained by several factors in the Hispanic/Latino population, including lack of access to health care and SARS-CoV-2 testing or medical insurance, less opportunity for social distancing with multigenerational or multi-family housing, the necessity of continuing to work in face-to-face settings where there is a high risk of transmission for financial or employment concerns, or higher disease prevalence [2, 9]. Disparities among Hispanic/Latinos persist for other diseases as well [10] and exacerbated by social determinants of health. Improved efforts to address structural barriers related to COVID-19 and healthcare access in general include accessible testing sites specifically in Hispanic/Latino communities, improved access to care, culturally competent messaging and healthcare settings, focused outreach and media and other interventions specifically adapted to this community. In this study, with a relatively smaller NH Black population, we also found the significantly higher rates of being tested positive among the NH Black population compared to the NH Whites, though the positivity rate in NH Black population has dropped below 10%. This may be more significant in other larger urban centres where there may be a larger NH Black population.

Limitations of this study include that it was a single study site and may not represent other settings in the United States. The positivity rate may depend on how many tests are done during that specific period of time. Our analysis involved trends based on reopening in Rhode Island which may differ by state. Despite this, we found that despite overall state trends, Hispanic/Latino populations may experience persistently higher rates of infection over time.

In conclusion, tailoring the policy to meet the needs of the Hispanic/Latino communities, and reinforcing COVID-19 mitigation approaches such as wearing face masks, physical distancing, hand washing and other measures may help further reduce the spread of SARS-CoV-2 infection in this population.

## Data Availability

The data that support the findings of this study are available on request from the corresponding author, C-H W. The data are not publicly available due to their containing information that could compromise the privacy of research participants.
